# B cell responses in older adults with latent tuberculosis: Considerations for vaccine development

**DOI:** 10.15761/GVI.1000112

**Published:** 2016-05-27

**Authors:** Sina Helbig, Sergey Rekhtman, Kristen Dostie, Alexander Casler, Thomas Schneider, Natasha S. Hochberg, Lisa Ganley-Leal

**Affiliations:** 1 Section of Infectious Diseases, Boston University School of Medicine, Boston, MA, USA; 2 Department of Epidemiology, Boston University School of Public Health, Boston, MA, USA; 3 Center for International Health Research, Rhode Island Hospital, Providence, RI, USA; 4 STC Biologics, Inc. Cambridge, MA, USA

## Abstract

Reactivation of latent tuberculosis (LTBI) is more common among the aging population and may contribute to increased transmission in long-term health care facilities. Difficulties in detecting LTBI due to potential blunting of the tuberculin skin test (TST), and the lowered ability of the elderly to tolerate the course of antibiotics, underscore the need for an effective vaccine. Immuno-senescence reduces the capacity of vaccines to induce sufficient levels of protective immunity against many pathogens, further increasing the susceptibility of the elderly to infectious diseases. We sought to evaluate the response of B cells to *Mycobacterium tuberculosis* (*Mtb*) in residents of long-term care facilities to determine the feasibility of using a vaccine to control infection and transmission from reactivated LTBI. Our results demonstrate that although B cell responses were higher in subjects with LTBI, *Mtb* antigens could stimulate B cell activation and differentiation *in vitro* in TST negative subjects. B cells from elderly subjects expressed high basal levels of Toll-like receptor (TLR)2 and TLR4 and responded strongly to *Mtb* ligands with some activation pathways dependent on TLR2. B cells derived from blood, tonsil and spleen from younger subjects responded similarly and to the same magnitude. These results suggest that B cell responses are robust in the elderly and modifications to a TB vaccine, such as TLR2 ligand-based adjuvants, may help increase immune responses to a protective level.

## Introduction

Latent tuberculosis infection (LTBI) has substantially declined in the developed world; however, reactivation of disease is prevalent among the aging population [1]. Tuberculosis (TB) in the elderly is thought to be primarily attributed to reactivation of LTBI; enhanced transmission within the nursing home setting has been reported [2]. Tuberculosis rates in the USA are thus highest among nursing home or long term care facility populations [3]. In addition, increasing rates of reactivation are facilitated by high prevalence in older adults with co-morbidities associated with progression to TB (e.g. chronic renal failure, poorly controlled diabetes) coupled with a higher proportion of the population living to an older age and being cared for in long-term care facilities [4]. Residents may also be subjected to crowding conditions further increasing transmission risk [5].

Primary infection with *Mycobacterium tuberculosis* (*Mtb*) leads to clinical disease in only about 10% of cases in the general population [6]. Thus in 90%, infection appears to be controlled by the immune response. However, complete eradication of the pathogen is observed in only about 10% of these individuals. In the remainder, *Mtb* is contained in a non-replicating state in the absence of clinical disease, which is referred to as LTBI. LTBI may develop into a fulminant disease upon deterioration of the immune system, such as in the state of AIDS or immuno-senescence [7,8]. A compromised immune system also results in poor detection rates of infection due to the tuberculin skin test (TST) reliance on a strong delayed-type hypersensitivity reaction [9]. Our recent report demonstrates that a discordance between TST and other diagnostics may add to the risk of transmission in nursing homes [10].

Elderly patients have higher rates of adverse events, notably hepatotoxicity, in response to current LTBI therapy and thus may not tolerate long-term treatment required to eliminate infection [11,12]. Development of new therapeutic interventions against reactivation of LTBI and *Mtb* transmission, such as a vaccination program, for older, long-term care facility residents may reduce the impact of LTBI and the threat of infection to staff, visitors and the greater community [5,13]. Despite early evidence of humoral responses, the relatively low protection rates mediated by the Bacillus-Calmette Guerin (BCG)-vaccine suggested that humoral immunity was not critical for preventing infection and attention was shifted to the development of potent antibiotics [14,15]. In light of the persistent TB burden worldwide primarily fueled by the human immunodeficiency virus (HIV) epidemic and development of *Mtb* drug resistance, investigators have re-visited the importance of humoral immunity for control strategies.

Studies on TB immunity have mainly focused on T cells; thus, there are few published reports on the role of B cells or antibody in human disease [16]. Opsonizing IgG1 and IgG3 have been shown to rise in active TB patients with severe pulmonary disease, suggesting that antibody may function to clear *Mtb* infection [15,17]. This is supported by other studies showing that *Mtb*-specific IgG significantly enhances the complement-induced killing of *Mtb in vitro* [18,19]. *Mtb*-specific antibody can interfere with macrophage adhesion and phagolysosome fusion, as well as regulate granuloma formation. In addition, passive protection with intranasal monoclonal IgA antibodies, though short-lived, was shown against early infection in the lungs in mice. Interestingly, high dose intravenous human immunoglobulin (IVIG) given to *Mtb*-infected mice decreased bacillary load in the lungs and spleen, suggesting an additional protective role of antibodies, despite the lack of demonstrated *Mtb*-specificity in IVIG [20].

B cells are multifunctional and can enhance T cell responses through antigen presentation and secretion of immuno-regulatory cytokines [21,22]. B cells are a constituent of inducible ectopic pulmonary lymphoid aggregates, which have been described in both murine and human lungs at the sites where *Mtb* is thought to be contained [23–25]. The aggregates contain germinal center (GC) B cells indicating that B cell differentiation and perhaps development of protective immunity takes place in these tertiary lymphoid tissues [23]. These B cell follicles may also potentiate protective inflammation. In B cell-deficient mice, aerosol challenge with *Mtb,* results in a significantly higher bacillary burden in the lungs, and impaired granuloma organization, which was reversible by adoptive transfer of B cells suggesting that B cells may have several functions in controlling TB [26].

With research focusing on generating a highly diversified immune response through vaccination [27], we sought to characterize B cell activation and differentiation in an elderly nursing home cohort with LTBI. We demonstrate the feasibility of inducing targeted protective immunity through vaccination; this method could be applied to nursing home residents to help protect against infection with *Mtb* and reactivation of LTBI.

## Methods

### Study participants

This study was approved by the Boston University Institutional Review Board. Subjects were recruited from three local long-term care facilities. A review of medical records was performed to identify HIV-uninfected residents with a history of a positive TST. Subjects were HIV negative based on chart review, but no active determination of HIV status was performed for this study. An HIV-negative, sex- and age-matched (within 5 years) comparison group with no documented positive TST was also recruited. The median age of the 37 participants was 78 years (range 58 – 98; Table 1).

Upon informed consent, personnel administered a standardized questionnaire to study participants, or their legal authorized representative, to obtain information regarding their health and personal history. 15 ml of venous blood were then collected into heparinized tubes for determination of T-SPOT.TB test (interferon-gamma release assay; herein IGRA; Oxford Immunotech, MA) and for the immunological assays described below. Following phlebotomy, a TST was administered on the volar aspect of the forearm and read after 48 h using the ball-point pen and ruler method. A positive TST was defined as an induration of ≥ 10 mm, in accordance with national guidelines. Any questionable result was confirmed by a second reader. TST positive subjects who also had a positive IGRA are referred to as LTBI herein. Subjects with a negative reading are referred to as TST-neg. Additional details of the study protocol have been previously published [28].

### Blood and lymphoid tissue from younger subjects

Heparinized whole blood samples (n=6) from younger subjects (up to age 50) were purchased from Research Blood Components (Boston, MA). Discarded surgical samples of tonsil (n=3) and spleen (n=1) were purchased from the National Disease Research Interchange, PA. Their history of TB, exposure to *Mtb*, or BCG vaccine was not disclosed.

### *Ex vivo* analyses in fresh whole blood

CD19^+^ B cells in fresh whole blood were assessed for *ex vivo* surface expression of CD27, CD36, TLR2 and TLR4. Neutrophils in fresh whole blood were assessed for TLR2 and TLR4 levels. 100 µl/tube of heparinized whole blood were incubated with fluorescently labeled antibodies (BD Pharmingen; eBioscience) at 4°C for 30 min. Red blood cells were lysed with 2 ml of FACS lyses buffer (BD Pharmingen) for 30 min at room temperature in the dark. Cells were washed with 2 ml of 0.2% bovine serum albumin (BSA) in PBS and evaluated by flow cytometry. Assessment of surface expression on B cells was performed with gates generated with anti-CD19 for each sample. Surface expression on neutrophils was performed using electronically-separated cells on FSC/SSC. The remaining whole blood was centrifuged; the plasma collected and stored at −20°C for the detection of antibodies, inflammatory mediators, and cytokines detailed below.

### Immunoassays to assess cellular responses to *Mycobacterium tuberculosis*

Tissues were snipped, gently homogenized and filtered through a 70 µ cell strainer to obtain a single cell suspension. Tissue cells and peripheral blood (PB) were subjected to Ficoll-Hypaque density gradients (Atlanta Biologicals, Lawrenceville, GA) to obtain mononuclear cells (MC). B cells were isolated from MC by negative bead isolation (Invitrogen). MCs were seeded at 2 × 10^6^ cells/ml and B cells at 1 × 10^6^ cells/ml in RPMI 1640 supplemented with 10% heat-inactivated fetal calf serum, 1 mM penicillin-streptomycin, and 2 mM l-glutamine (Invitrogen). The following reagent was obtained through BEI Resources, NIAID, NIH: *Mycobacterium tuberculosis*, Strain H37Rv, Whole Cell Lysate (WCL), NR-14822. Cells were stimulated with medium alone, 2, 5, 10 or 20 µg H37Rv WCL; anti-CD40 (1 µg/ml; R&D Systems), *E. coli* LPS (0.1 or 1 µg/ml), or PAM3CSK4 (1 µg/ml) (both from Invivogen). In some experiments, blocking antibody to TLR2 and TLR4 was included at 10 µg/ml (eBioscience) At 24, 48 and 72 hrs, cell-free supernatants were collected and stored at −20°C until use for measurement of secreted cytokines. The cells were harvested and processed for flow cytometry for B cell activation and differentiation markers, including CD19, CD69, TLR2, TLR4, CD86, CD95, CD77, CD38, and CD27 with antibodies purchased from BD Pharmingen and eBioscience. Cells were fixed with 0.2% paraformaldehyde (Electron Microscopy Sciences, Washington, PA) prior to evaluation by flow cytometry.

### Measurement of cytokines and *Mycobacterium*-specific antibodies

Cell-free culture supernatants were evaluated for levels of IFN-γ, IL-10, IL-4 and IL-8 by commercially available ELISA reagents (R&D Systems, Minneapolis, MN). Cytokines IL-6 and IL-8 were measured in plasma by ELISA (R&D Systems). Plasma antibody was assessed for *Mycobacterium*-specific antibody by ELISA. Plates were coated with 5 µg/ml of WCL in PBS overnight at 4°C. Plates were washed and blocked with 1% BSA/PBS for 1 hour. Plasma was applied at a dilution of 1:80 following titration of plasma (see Figure 2A) and incubated for 4 hours at room temperature. *Mtb*-specific IgG1, IgG2, IgG3 and IgG4 were detected with HRP-conjugated secondary antibodies purchased from Southern Biotech.

### LPS and BPI

To detect potential subclinical bacterial infection that would affect baseline measures of cellular activation, levels of LPS/endotoxin (LAL; Lonza) and bactericidal permeability increasing protein (BPI; Hycult Biotech ELISA) were measured in the plasma.

### Assessment of Toll-like receptor ligands in WCL

TLR2- and TLR4-expressing HEK293 cells were purchased from Invivogen and cultured per manufacturer’s instructions. Once TLR2 and TLR4 surface expression was confirmed by flow cytometry, cells were seeded at 50,000 cells/well in 96 well plates. Cells were treated with titrated concentrations of WCL. *E coli* LPS and Pam3CSK4 were used as controls. The next day, supernatants were collected for measurement of IL-8 by ELISA.

### Statistical analyses

Statistical analyses were performed using GraphPad Prism (GraphPad Software). A one-way analysis of variance with Dunn’s post-test and the Mann-Whitney *U* test were used for multiple- or single-group comparisons, respectively. Possible correlations were examined using Spearman’s rank correlation test. Group sample sizes differ among the tests because some patient samples were unavailable.

## Results

### Baseline *ex vivo* B cell activation levels in LTBI subjects

**Figure 1:**
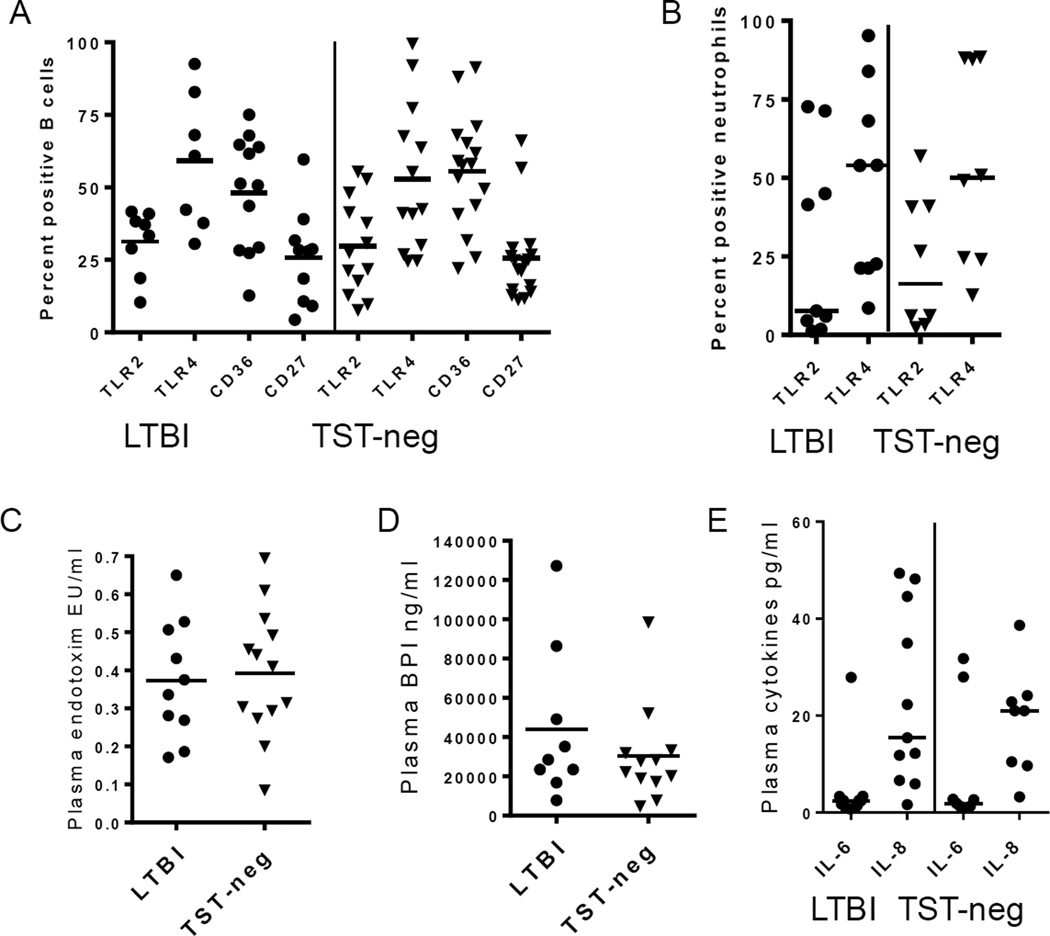
Baseline levels of inflammatory indicators are low in LTBI A. *Ex vivo* levels of B cell receptors involved in bacteria recognition were similar in LTBI and TST-neg subjects. Percentages of CD27^+^ B cells are also similar. B. *Ex vivo* levels of TLRs on neutrophils were similar between LTBI and TST-neg subjects. C. Plasma levels of endotoxin were similar between LTBI and TST-neg subjects. D. Plasma levels of BPI were also similar between LTBI and TST-neg. subjects. E. There were no differences in the plasma levels of IL-6 and IL-8 between LTBI and TST-neg subjects. Horizontal lines indicate medians. All differences in means between LTBI and TST-neg are *P*>0.05.

Our goal was to determine the ability of B cells from elderly subjects to respond to *Mtb* antigens as a proxy for vaccine responsiveness. Baseline expression of receptors involved in microbial recognition on B cells in fresh whole blood was measured in the two groups. Surface levels of TLR2, TLR4, and CD36 were high on circulating B cells in the elderly subjects compared to previously published data on cells from adult, non-elderly populations [29]. However, there were no differences between LTBI and TST-neg groups (Figure 1A). There were no differences in the levels of circulating memory B cells as measured by percentages of CD19+ cells expressing CD27 (Figure 1A).

### Inflammatory biomarkers are similar between LTBI and TST-neg

To determine if a systemic inflammatory response existed that might affect cellular response *in vitro*, we measured surface levels of TLR2 and TLR4 on neutrophils as a proxy indicator and found no difference between the two groups (Figure 1B) [30].

LPS/endotoxin levels in the bloodstream may also serve as a measure for subclinical infection [31]. Plasma endotoxin levels were similarly low in both groups (Figure 1C). Likewise, concentrations of BPI, a molecule that is released from circulating neutrophils in response to LPS, were low in both groups (Figure 1D) [32].

IL-6 and IL-8, pro-inflammatory cytokines described to be elevated in active TB, were equally low in both groups (Figure 1E) [33]. Overall, these data suggest that the baseline pro-inflammatory state is similar between the two groups. Thus, there were no systemic inflammatory measures identified that would confound the results obtained *in vitro*.

### *Mtb*-specific memory responses in LTBI

**Figure 2:**
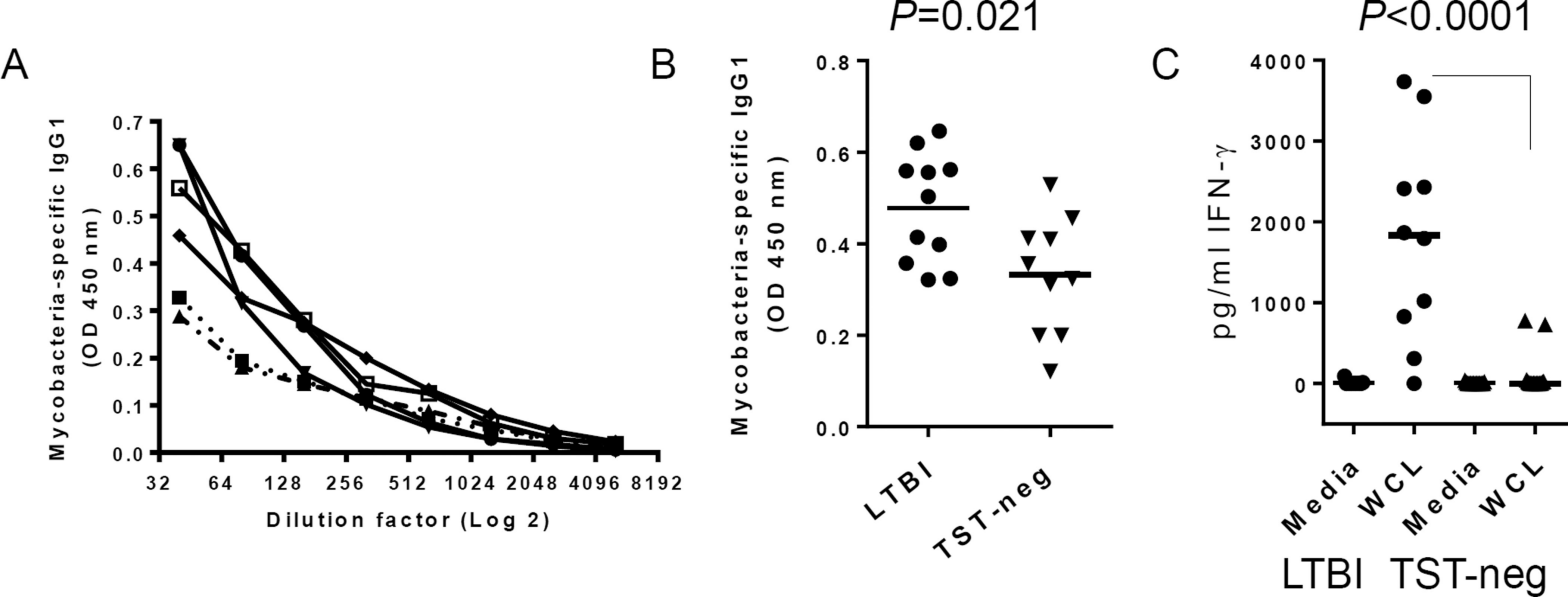
Evidence of pre-existing immunity to *Mtb* in LTBI A. Titration of *Mtb*-specific IgG1 in 5 LTBI subjects by ELISA. B. Mean OD values of *Mtb*-specific IgG1 at 1:80 plasma dilution are higher in LTBI subjects. C) IFN-γ secretion in response to WCL of *Mtb* is higher in LTBI. PBMCs were incubated for 72 hours and IFN-γ measured by ELISA. Horizontal bars indicate medians.

The level of *Mtb*-specific IgG was measured in plasma as an indicator of pre-existing immune responses and a gauge for the presence of antigen-specific memory B cells (Figure 2A). A significantly higher concentration of *Mtb*-specific IgG1 was detected in the plasma of LTBI subjects compared to TST-negative subjects (Figure 2B). Levels of antigen-specific IgG2 or IgG3 were equally low in both groups and there was no statistical difference between LTBI and TST- negative (data not shown). *Mtb*-specific IgG4 was not detected in either group (data not shown).

PBMCs from subjects with LTBI secreted significantly more IFN-γ, but not IL-10, in response to WCL of *Mtb* (Figure 2C; shown are IFN-γ levels). Thus, most TST-identified LTBI subjects had indicators of pre-existing memory immune responses to *Mtb*.

### B cells from elderly subjects are activated in response to *Mtb* antigens *in vitro*

**Figure 3:**
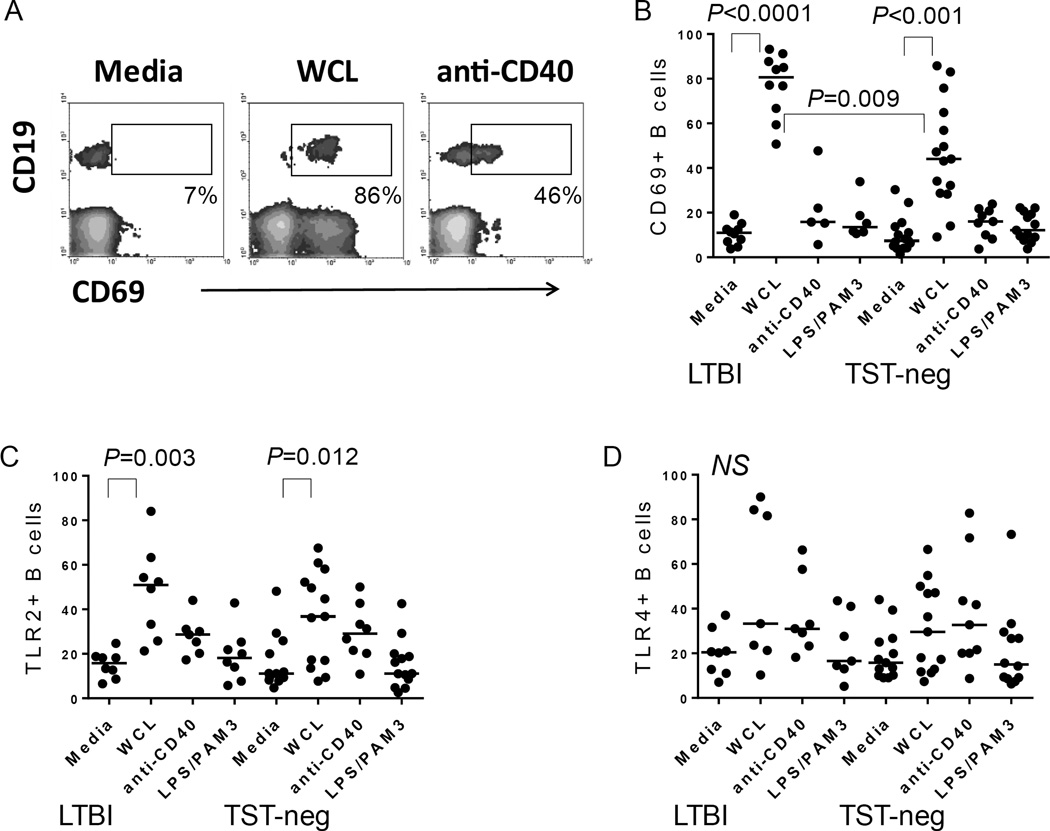
*Mtb* antigens activate B cells A. Flow cytometric plot depicting the level of CD69 expressed by CD19^+^ B cells in PBMC cultures. Shown are plots from an LTBI subject. WCL also induced CD69 expression by non-B cells as reported in Hochberg et al., 2016 [3]. B. Composite data demonstrating an increase in CD69^+^ B cells following treatment with the stimuli indicated on figure. WCL from *Mtb* increased B cell expression of CD69, whereas anti-CD40 or a combination of *E. coli* LPS (TLR4 ligand) and PAM3CSK4 (TLR2 ligand) did not. C. Composite data demonstrating an increase in TLR2^+^ B cells following treatment with the stimuli indicated on figure. WCL from *Mtb* increased B cell expression of TLR2 in both groups. D. In contrast, TLR4 is not upregulated significantly by any of the stimuli tested in either group. Horizontal bars indicate medians.

PBMCs treated with WCL of *Mtb* for 72 hours demonstrated a high level of activated B cells as measured by CD69 expression, a general activation marker (Figure 3A & 3B). LTBI was associated with higher percentages of activated B cells after stimulation compared to TST-neg (Figure 3B).

Although elderly subjects demonstrated high basal levels of TLR2+ B cells, we found that treatment of PBMCs with WCL resulted in further increased levels of TLR2 expression by B cells in both LTBI and TST-neg compared to untreated cells (Figure 3C). In contrast, levels of TLR4 did not rise considerably in response to WCL and there was no difference between the groups (Figure 3D).

As a control for the non-specific stimulation of B cells by WCL in the mixed cell culture, PBMCs were stimulated with a combination of Pam3CSK4, a TLR2 ligand, and *E. coli* LPS, a TLR4 ligand. This stimulation did not induce significant activation of B cells compared to untreated cells from the elderly groups (Figure 3B–3D).

### B cell differentiation in response to *Mtb* antigens is intact in older adults

**Figure 4:**
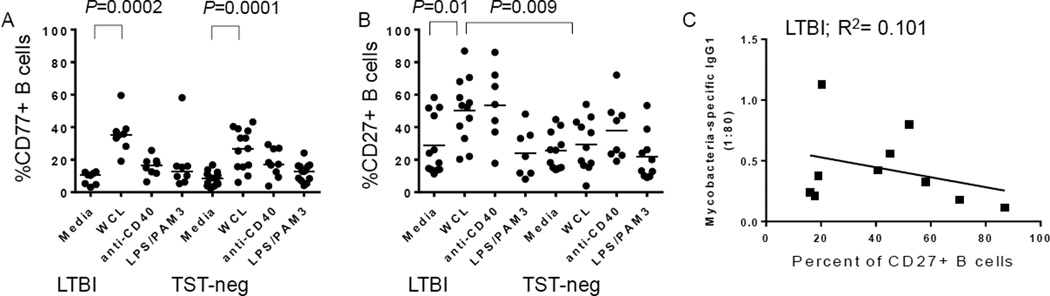
Mycobacteria antigens stimulate B cell differentiation from older subjects A. Upregulation of CD77^+^ B cells following treatment with the stimuli indicated on figure. WCL from *Mtb* increased B cell expression of CD77 in both groups, whereas anti-CD40 and a combination of PAM3CSK4 and *E. coli* LPS did not. B. CD27 levels changed in response to WCL from *Mtb* only in LTBI. Horizontal bars indicate medians. C. Plasma levels of *Mtb*-specific IgG1 had an inverse, but statistically insignificant, relationship with percentages of CD27+ B cells following WCL treatment.

B cells differentiate in lymphoid germinal centers (GC) and transition through several stages of activation as they develop into memory B cells or plasma cells [34]. We assessed expression of CD77 as a marker for GC B cell development in response to WCL. B cells from LTBI subjects expressed a higher level of CD77 in response to WCL compared to untreated cells (Figure 4A). Importantly, B cells from TST-neg expressed CD77 in response to *Mtb* antigens as well (Figure 4A).

B cells from LTBI, but not those from TST-neg control subjects, had increased levels of the memory marker, CD27, in response to WCL (Figure 4B). Because the higher level of *Mtb*-specific IgG1 suggested higher pre-existing levels of *Mtb*-specific memory B cells in LTBI, we assessed the relationship between WCL-induced CD27 expression and the level of *Mtb*-specific IgG1. An inverse, but not statistically significant, relationship between increased CD27+ B cells and *Mtb*-specific IgG1 suggests that the existing *Mtb*-specific memory B cells may follow a different pathway of differentiation upon encounter with infection, such as re-entering the GC (Figure 4C). However, there was no correlation between *Mtb*-specific IgG1 levels and a change in CD77 levels in response to WCL (data not shown).

### B cell activation markers correlate with intensity of diagnostic test readouts

**Figure 5:**
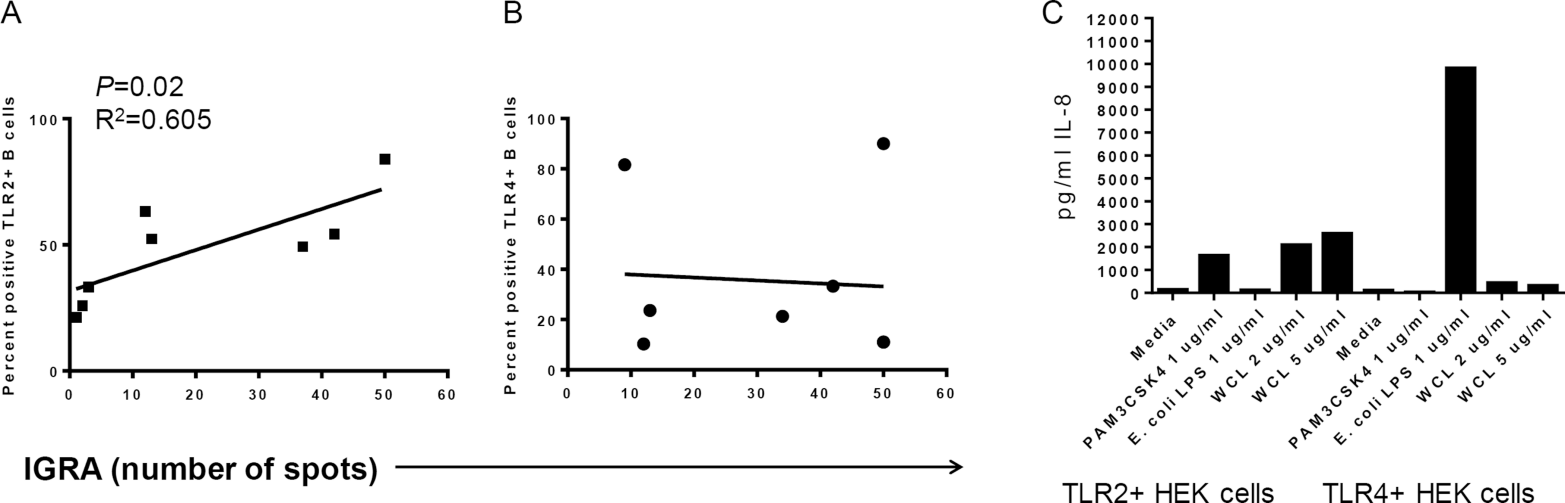
*Mtb*-mediated upregulation of TLR on B cells correlates with IGRA intensity A) Percentages of TLR2^+^ B cells following PBMC culture with WCL from *Mtb* for 72 hrs correlated with the intensity of IGRA diagnostic. B) In contrast, percentages of TLR4^+^ B cells following PBMC culture with WCL did not correlate with the intensity of IGRA. C. TLR2^+^ HEK cells respond to WCL by secreting IL-8 (left side of plot), but there was a weak response from TLR4+ HEK cells to WCL (right side of plot.)

The TST and IGRA are largely T cell-mediated responses, but B cells have been shown to have roles in granuloma formation in TB, which is a similar delayed-type hypersensitivity reaction as the TST. B cell activation, including upregulation of TLR2 and CD69, correlated with the intensity of both the TST (diameter size) and the number of spots from IGRA (Figure 5A; shown are TLR2 levels). However, there was no relationship with the upregulation of TLR4 (Figure 5B). This observation suggests that TLR2 responses by B cells may play a role in *Mtb*-mediated immunity.

Although WCL likely contains multiple antigens that cross-link B cell receptors, culture of TLR2+ HEK and TLR4+ HEK cells with WCL from *Mtb* demonstrate the existence of TLR2 ligands and weak TLR4 ligands (Figure 5C).

### Mononuclear cells from uninfected, younger subjects respond similarly to WCL

**Figure 6:**
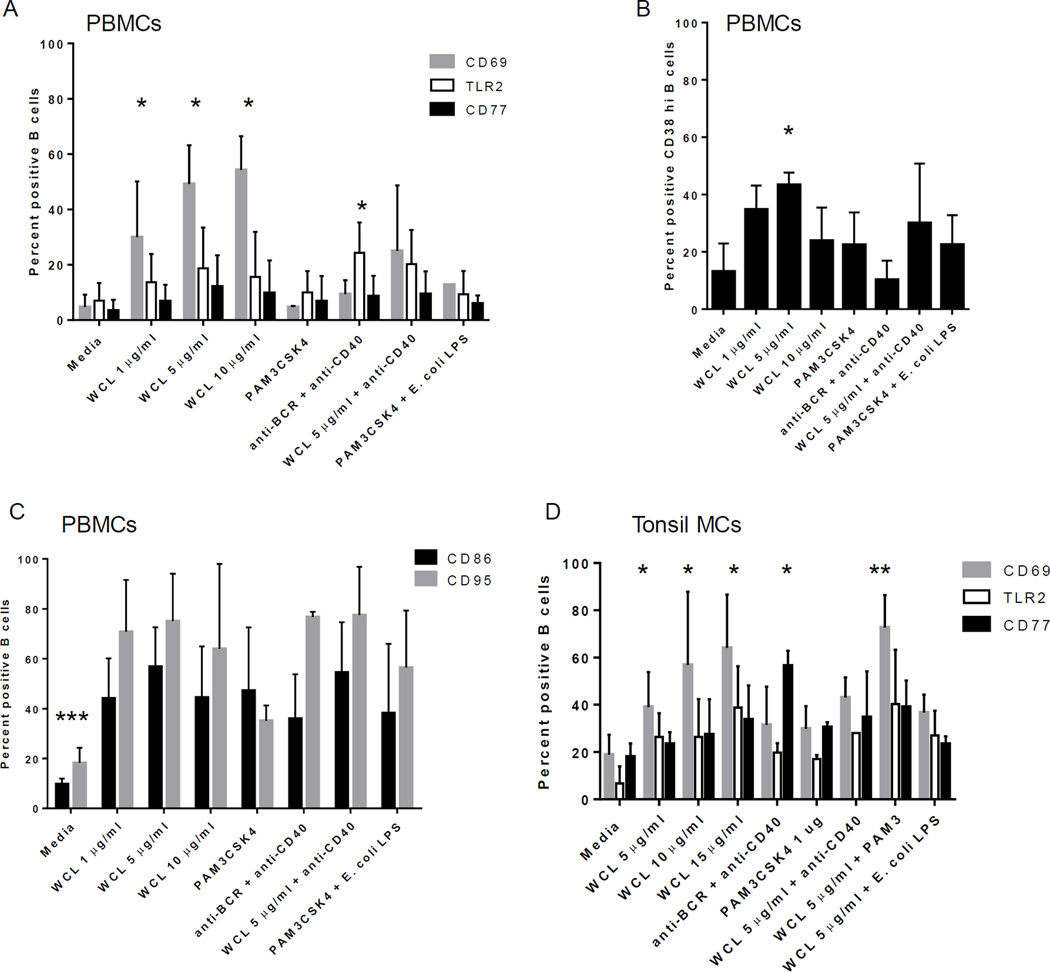
B cells from non-elderly subjects respond similarly to those from elderly subjects A. B cells within PBMCs express CD69, TLR2 and CD77 as well as CD38 (B) in response to WCL following culture; *indicates a statistically significant difference in means from media alone; *P*<0.02. C. B cells within PBMCs express additional markers of activation, including CD86 and CD95 in response to WCL. A combination of Pam3CSK4 and *E. coli* LPS also induced CD86 and CD95. **indicates means of stimuli-induced activation differed from media alone; *P*<0.02. D) Mucosal B cells demonstrate similar responses to WCL and upregulate CD69, TLR2 and CD77. *indicates a statistically significant difference compared to media alone; P<0.02. All data shown is at the 72 hour time point and n=3 separate experiments.

To determine if the activation level of B cells to *Mtb* antigens from the elderly subjects was muted, we assessed responses in PBMCs (<50 years) from younger subjects with an unknown history of TB, exposure to *Mtb or* BCG vaccine. In general, the cellular response to *Mtb* antigens was similar, and of the same intensity and kinetics, as those found in the elderly cohorts, including the upregulation of CD69, TLR2 and CD77 at 72 hours (Table 2; Figure 6A). Likewise, TLR4 was not significantly upregulated (data not shown). WCL induced the upregulation of CD38^high^ B cells, which may represent GC or plasma cell precursors (Table 2; Figure 6B). In addition, CD86 and CD95 were increased B cells within PBMCs demonstrating that human B cells become highly activated and differentiate in response to *Mtb* antigens, even in the naive state (Table 2; Figure 6C). Curiously, there was little upregulation of these markers at 24 hours of culture (data not shown).

B cells within mononuclear cells from mucosal and systemic lymphoid tissues, including tonsil and spleen, were also assessed. Lymphoid B cells responded strongly to WCL by upregulating CD69, TLR2, TLR4, and CD77 by 72 (but not 24) hours of treatment (Table 2; Figure 6D). Thus, while human B cells recognize WCL of *Mtb*, the inherent delay in cellular activation may indicate the presence of B cell immuno-evasive antigens.

### WCL of *Mtb* directly activates human B cells

**Figure 7:**
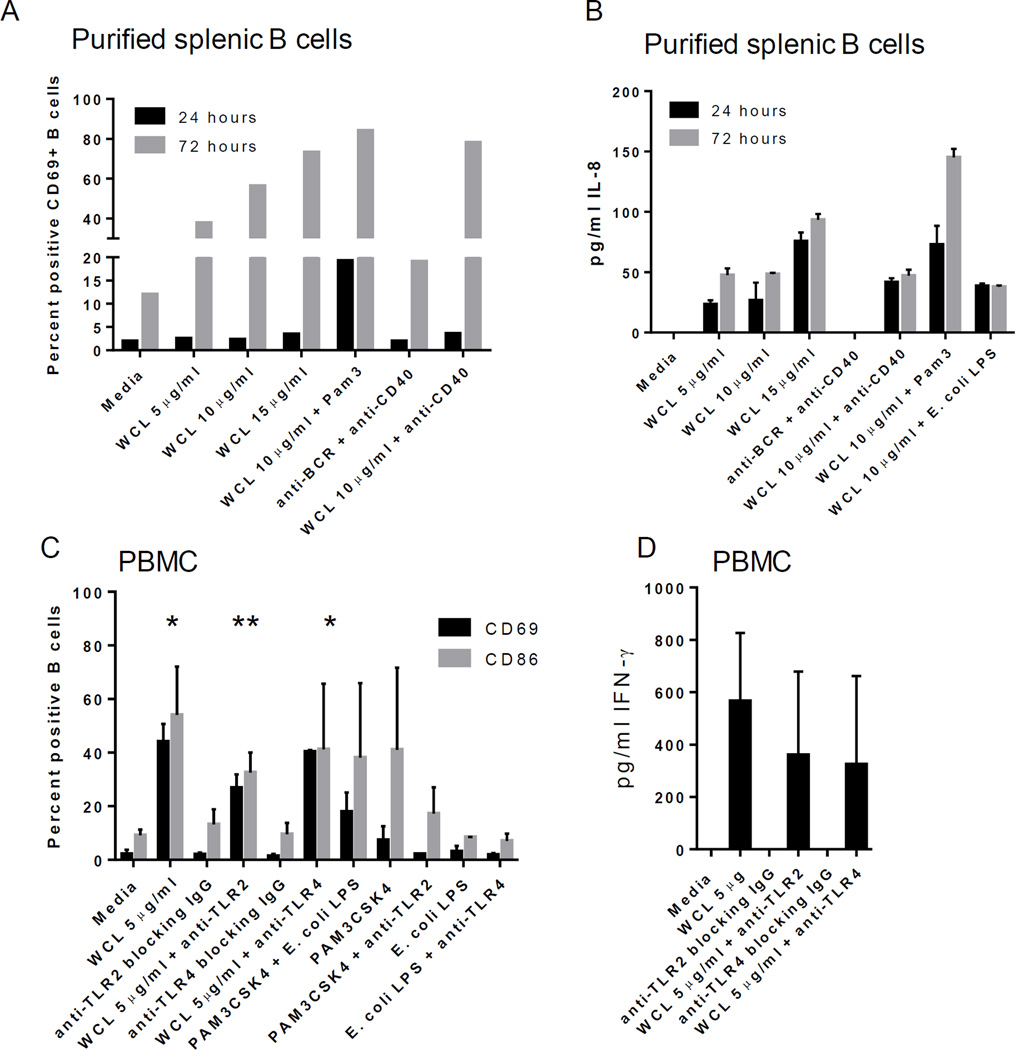
Direct response of purified B cells is enhanced by TLR2 ligand A) Purified splenic B cells responded similarly to WCL as those within mixed cell cultures, including a delay in CD69 expression. This delay is reduced by the addition of TLR2 ligand, Pam3CSK4 (black bar.) B) Purified splenic B cells secrete IL-8 in response to WCL, which is enhanced by the addition of Pam3CSK4 noted by 72 hrs. C) B cells within PBMC require TLR2, but not TLR4, for optimal activation in response to WCL; *indicates statistically significant differences in means compared to media alone for both CD69 and CD86; **indicates anti-TLR2 reduced the response to a statistically significant difference in mean compared to WCL alone; 72 hr time point. D) IFN-γ secretion was modestly reduced with both anti-TLR2 and anti-TLR4 blocking antibody; 72 hr time point.

To determine the direct effect of *Mtb* antigens, purified B cells from tonsil and splenic mononuclear cells were cultured with WCL. Expression of activation markers were induced by WCL, but demonstrated a delay at 24 hours (Table 3; Figure 7A; shown are CD69^+^ B cells). WCL also directly induced IL-8 secretion by purified B cells (Figure 7B).

### *Mtb*-induced B cell activation is partially dependent on TLR2

We next assessed if TLR2 or TLR4 played a role in B cell activation based on our above observations. The addition of a TLR2 ligand (Pam3CSK4) to PBMCs or tonsil MC treated with WCL increased B cell expression of CD69 and CD86 (Figures 6D; tonsil MC; Figure 7B; purified splenic B cells). Furthermore, the delay in B cell activation was reduced with the addition of Pam3CSK4, but not *E. coli* LPS, to WCL (Figure 7A and 7B). In contrast, addition of TLR4 ligand, *E. coli* LPS had no effect (data not shown).

Blocking antibody to TLR2 reduced the expression of these activation markers in response to WCL (Figure 7C); however, B cell differentiation, as measured by CD38, was not affected (not shown). In contrast, blocking TLR4 had no influence on any of the B cell activation or differentiation markers measured (Figure 7C).

### Cytokine production is not dependent upon TLR2 or TLR4

To determine the mechanism by which IFN-γ memory may be developed, PBMCs were treated with WCL in the presence of anti-TLR2 or anti-TLR4 blocking antibodies. Both antibodies had a modest effect on the production of IFN-γ *in vitro* (Figure 7D). In contrast, IL-8 secretion was not affected by either anti-TLR2 or anti-TLR4 within PBMC or MC from tissues (data not shown).

## Discussion

Older adults are at high risk for TB and transmission of *Mtb* has been reported in long-term care facilities for the elderly [4,35]. Because age can affect immune responses required for effective diagnosis and vaccination, improved tools, such as efficacious vaccines, to help control the disease are critically needed [28]. Further, the protective mechanisms involved in containing mycobacteria are poorly defined and the roles of B cells or antibodies in controlling TB should be clarified to improve vaccine strategies [36].

Recent reports suggest that an important site of B cell activation and differentiation in TB is within pulmonary ectopic lymphoid aggregates [23]. Bronchus-Associated Lymphoid Tissue (BALT) develops in peribronchial, perivascular and interstitial areas of the lung in response to infection, but it is not normally present in healthy human lungs [37]. BALT is characterized by activated and GC B cells supported by a central follicular dendritic cell network, similar to typical lymphoid tissues [37]. Ectopic lymphoid aggregates in TB are thus similar to BALT and situated adjacent to tuberculoid granulomas. These aggregates likely contain GC-like B cells, but have not been well-characterized in humans. Patients who lacked these lymphoid aggregates had higher numbers of mycobacteria in their sputum suggesting a role for containment of *Mtb* [23]. Interestingly, no reactivation of TB was observed in Rituximab (which does not eliminate IgA-specific B cells)-treated rheumatoid arthritis patients with LTBI suggesting that *Mtb*-specific IgA may be a critical effector antibody isotype in the lung [38]. B cells may also play roles in recruiting cells to the lungs through secretion of chemokines or inducing the activation of T cells. With a new focus on generating a highly diversified immune response through vaccination, our goal was to define the effect of WCL of *Mtb* on B cells in the elderly to help determine the feasibility of vaccination strategies to control activation and transmission in LTBI [27].

Although we did not measure IgA, we demonstrate that plasma from the LTBI elderly population contains increased *Mtb*-specific IgG1 levels, suggesting that pulmonary *Mtb* in LTBI stimulates the humoral arm of the immune system. We report that peripheral blood B cells from LTBI and TST-neg elderly subjects respond strongly, and similarly, to *Mtb.* The magnitude of the response from circulating and mucosal B cells from younger subjects was comparable. In general, our data demonstrate that B cells upregulate surface molecules associated with activation (CD69), microbial recognition (TLR2 and TLR4) and differentiation (CD38, CD77, CD95) as well as those involved in T cell activation (CD86). Most of these markers of activation and differentiation were increased to a higher degree on B cells from LTBI subjects. Only B cells from LTBI upregulated CD27 in response to *Mtb* antigens, a marker of memory B cells. Nevertheless, increased B cell activity from TST-negative subjects in response to *Mtb* indicates the potential to induce a potent immune response through vaccination in elderly patients as evidenced by germinal center B cell differentiation in the two cohorts.

Interestingly, our data suggest that there may be immunosuppressive antigens directed at B cells contained within the WCL of *Mtb* [39]. The addition of a TLR2 ligand adjuvant may circumvent some of this effect in a vaccine strategy. We have shown previously that IFN-γ does not play a direct role in TLR2 expression by B cells, but the significance of TLR2-mediated B cell response in LTBI may be reflected in the positive relationship between IGRA and TLR2 upregulation with *Mtb* antigens [40]. Thus, we hypothesize that a recall response may involve TLR2+ B cells in bacterial containment. This is supported by our findings that TLR2 plays a role in B cell activation in response to WCL *in vitro*. Activation of B cells via TLR2 in a vaccine strategy may better prepare the immune system to clear an infection. In contrast, *E. coli* LPS and other TLR4 ligands have historically been a poor stimulator of human B cells, despite elevated levels of TLR4 in this cohort [41,42]. TLR2 has been shown to drive the development of memory B cells, which appear to retain surface expression of TLR2 imprinting the cell with the ability to rapidly respond to pathogens [43]. We and others have also demonstrated that TLR2+ B cells isolated from mucosal lymphoid tissues have characteristics of GC B cells and that TLR2-stimulated B cells secrete copious amounts of chemokines, which may recruit essential cells in the case of *Mtb* exposure [44,45]. Finally, TLR2 may play a role in directing B cells to mucosal tissues by inducing the upregulation of specific homing receptors [46].

In conclusion, a mixed *Mtb* antigen vaccine containing exogenous TLR2 ligands may induce strong protection against reactivation of LTBI or infection with *Mtb* [27,47]. Addition of TLR2 ligands to a vaccine may overcome the potentially inherent immuno-evasive antigens and induce B cell activation and homing to the lung upon infection or reactivation of LTBI [39]. Future studies should focus on better characterization of the role that BALT plays in generating protective immunity.

## Figures and Tables

**Table 1:** Characteristics of subjects.

	Cases (n=12)	Controls (n=14)

Median age, years (range)	78 (60–92 range)	79 (58–98 range)

Male, n (%)	11 (92)	10 (71.4)

*Race*(%)		
Black non-Hispanic	9 (75)	8 (61.5)
Black Hispanic	1 (8.3)	0
White non-Hispanic	2 (16.7)	3 (23.1)
White Hispanic	1 (8.3)	2 (15.4)

Median BMI (range)	23.8 (19.2–30.5)	27 (16.5–33)

US-born, n (%)	2 (16.7)	7 (50)

Median years in nursing home (range)	5 (1–19)	5.5 (0–18)

Median number of roommates (including self)	3	4

Daily eating in communal dining room (% citing yes)	8 (66.7)	9 (64)

All P=NS

**Table 2:** Effect of WCL of *Mtb* on splenic B cells within MC. 72 hr culture.

	%CD69	%TLR2	%TLR4	%CD77	%CD38	%CD27
Media	45.9	18.1	13.6	13.2	25.1	42.0
WCL 5 µg/ml	65.6	69.1	63.1	23.6	62.5	62.0
WCL 10 µg/ml	66.2	74.7	66.1	24.2	59.7	63.2
WCL 15 µg/ml	68.4	71.1	66.3	24.5	51.1	60.8
anti-BCR + anti-CD40	79.8	66.3	60.2	17.7	78.4	50.7
WCL 10 µg/ml + anti-CD40	66.6	83.9	76.1	28.0	68.4	66.2
WCL 10 µg/ml + Pam3	71.1	85.7	81.1	34.6	75.8	71.3
WCL 10 µg/ml + *E. coli* LPS	74.3	78.5	68.9	23.4	71.9	62.8

**Table 3:** Effect of WCL of *Mtb* on purified splenic B cells. 72 hr culture.

	%TLR2	%TLR4	%CD77	%CD38 hi	%CD27
Media	11.1	9.9	9.1	7.6	24.6
WCL 5 µg/ml	42.5	18.1	10.9	46.3	37.5
WCL 10 µg/ml	21.2	30.6	9.6	20.7	42.5
WCL 15 µg/ml	24.3	48.1	7.8	10.1	43.9
anti-BCR + anti-CD40	9.8	12.1	6.3	7.2	22.4
WCL 10 µg/ml + anti-CD40	23.8	50.8	5.6	13.5	44.9
WCL 10 µg/ml + Pam3	31.7	58.4	9.7	14.1	48.1
WCL 10 µg/ml + *E. coli* LPS	24.2	46.3	4.9	10.9	43.1
